# Inhibition of USP7 suppresses advanced glycation end-induced cell cycle arrest and senescence of human umbilical vein endothelial cells through ubiquitination of p53: USP7 inhibits ubiquitination of p53

**DOI:** 10.3724/abbs.2022003

**Published:** 2022-02-21

**Authors:** Xu Li, Tao Wang, Yue Tao, Xiaojun Wang, Limeng Li, Jianjun Liu

**Affiliations:** Department of Vascular Surgery Qingpu Branch of Zhongshan Hospital Fudan University Shanghai 201700 China

**Keywords:** diabetic foot, USP7, p53, cell senescence, deubiquitination

## Abstract

Diabetes mellitus is a n arising public health concern, and diabetic foot is one of the most common complications of diabetes. Current management for diabetic foot cannot reach optimal remission. In this study, we aim to explore the mechanism underlying the pathogenesis of diabetic foot and provide novel strategies for the treatment of diabetic foot. A total of 10 normal skin tissues and 20 diabetic foot ulcer specimens are collected. Cell proliferation is determined by CCK-8 assay. Cell cycle is determined by flow cytometry, and cell senescence is evaluated by β-galactosidase staining. Co-immunoprecipitation assay is used to explore the interaction between USP7 and p53. Advanced glycation end products (AGEs) are used to establish diabetic cell model, and streptozotocin (STZ) is used to establish diabetic rat model. Our results showed that USP7 expression is increased in diabetic foot ulcer and in human umbilical vein endothelial cells (HUVECs) after treatment with AGEs. Inhibition of USP7 can reduce cell cycle arrest and cell senescence in HUVECs. Moreover, USP7 can interact with p53 and promote its expression through mediating its deubiquitination. Knockdown of p53 can reverse USP7-mediated cell cycle arrest and cell senescence in HUVECs. In diabetic rats, HBX 41108, the specific inhibitor of USP7, can significantly accelerate wound healing. Our study reveals that the inhibition of USP7 can suppress AGEs-induced cell cycle arrest and cell senescence of HUVECs through promoting p53 ubiquitination. USP7 is a potential target for the treatment of diabetic foot ulcers.

## Introduction

Diabetes mellitus is an arising public health concern with increasing prevalence around the world. Uncontrolled diabetes leads to peripheral neuropathy and peripheral arterial disease, which results in various complications [1]. Diabetic foot is one of the most common complications of diabetes, in which ischemia mediated by peripheral arterial disease is the key contributor of diabetic foot ulcers [2]. In conventional wound healing, inflammation can remove pathogens and cell debris, then growth factors and extracellular matrix are orchestrated to fill the wound area [3]. However, in diabetic foot ulcer, consistent inflammation impairs the formation of new tissues and blood vessels [4]. Angiogenesis is critical for wound healing and is regulated by various angiogenic factors. In diabetes, hyperglycemia can decrease the number of endothelial progenitor cells, which impairs angiogenesis [5]. In addition, hyperglycemia will elevate the level of advanced glycation end products (AGEs), which can suppress angiogenesis and wound healing [6]. Therefore, approaches to combat AGE-mediated effects appear to be promising therapeutics for diabetic foot ulcers.

Age and obesity are main risk factors of diabetes and are correlated with increased level of senescent cells, indicating the association between cell senescence and diabetes. Cell senescence is characterized by growth arrest in response to cell stress [7]. A genetic marker of cell senescence, CDKN2A, is implicated to increase the risk of diabetes and cardiovascular disease [8], and the senescence of adipose tissue can result in insulin resistance and type 2 diabetes [9]. Moreover, p53 is commonly known as a tumor suppressor and is also reported to regulate adipogenesis and cell senescence. The activation of p53 can prevent the differentiation of normal adipose, reduce insulin-induced glucose transportation, and increase lipolysis, which contributes to the inflammation and insulin resistance in adipocytes [10]. p21 is the downstream of p53 and is shown to regulate cell senescence [11]. However, the association between p21 and cell senescence in diabetes remains unclear.

Ubiquitination is a common type of post-translation modification. The ubiquitination process relies on three protein families: ubiquitin-activating enzyme (E1), ubiquitin-conjugating enzyme (E2), and ubiquitin ligases (E3) [12]. E1 is an ATP-dependent enzyme which initiates ubiquitination, then E2 transfers ubiquitin from E1 to E2, and E3 is responsible for the final step of ubiquitination through interacting with E2 and substrate proteins. The ubiquitination will lead to the degradation of proteins [ 13– 15]. In contrast, deubiquitination enzyme can remove ubiquitin from substrate proteins to prevent their degradation. Ubiquitin has been implicated in various diseases. A previous study indicated that E3 ubiquitin ligase MG53 is associated with the maintenance of metabolic homeostasis [16]. Moreover, the E3 ubiquitin ligase STUB1 could promote the ubiquitination of BMAL1 to alleviate cell senescence [17]. Therefore, ubiquitination may be associated with diabetes and cell senescence.

USP7 is a deubiquitinating enzyme and is implicated to correlate with glucose metabolism and insulin signaling [18]. However, its role in diabetic foot ulcers remains unclear. Herein, we aimed to explore the role of USP7 in diabetic foot ulcers and provide novel strategies for the treatment of diabetic foot ulcers.

## Materials and Methods

### Clinical specimens of diabetic foot ulcer

A total of 20 skin tissues of patients with diabetic foot ulcer were collected in Qingpu Branch of Zhongshan Hospital (Shanghai, China), which included 10 lesions with 1–2 diabetic ulcer severity score (DUSS) and 10 lesions with 3–4 DUSS. The control group was composed of 10 normal skin tissues in patients with trauma. Written informed consents were obtained from all patients. This study was approved by the Ethics Committee of Qingpu Branch of Zhongshan Hospital.

### Cell culture and cell transfection

HUVECs (American Type Culture Collection, Manassas, USA) were used in this study. Cells were cultured in DMEM (Gibco, Carlsbad, USA) with 10% fetal bovine serum (FBS). To modulate the expressions of USP7 and p53, three short hairpin RNAs (shRNAs) were designed and cloned into linearized pLKO.1 plasmid (Addgen, Watertown, USA). The sequences of shRNAs were shown as follows: shUSP7#1, 5′-GGTGTGAAATTCCTAACAT-3′; shUSP7#2, 5′-GCAGTTGGTGGAGCGATTA-3′; and shUSP7#3, 5′-CCAGGTGGACATAGACAAA-3′; shP53#1, 5′-GGAAGACTCCAGTGGTAAT-3′; shP53#2, 5′-GCGCACAGAGGAAGAGAAT-3′; and shP53#3, 5′-CCACTGGATGGAGAATATT-3′. To elevate the expression of USP7, the coding sequence of *USP7* was synthesized and cloned into pLVX-Puro plasmid (Clontech, Mountain View, USA). The recombinant plasmids together with the packaging plasmids psPAX2 and pMD2G were co-transfected into 293T cells using Lipofectamine 2000 (Invitrogen, Carlsbad, USA). At 48 h after transfection, recombined vectors were collected and used for transducing cells. Cells that were transduced with pLKO.1-scramble shRNA plasmid(shNC; 5′-GCCGACTATGGTAGAGAAT-3′) or blank pLVX-Puro plasmid (vector) were used as the negative controls.

### Experimental grouping

Treatments were divided into three groups as follows: in Group 1, HUVECs were treated with different concentrations of AGEs (125, 250, and 500 μg/mL) for 12, 24, or 48 h; in Group 2, HUVECs were treated with USP7 inhibitor HBX 41108 (5 μM) or transduced with shUSP7 lentivirus for 24 h, followed by the application of 250 μg/mL AGEs for 24 h; and in Group 3, HUVECs were transduced with oeUSP7 lentivirus and shP53 lentivirus.

### Cell counting kit (CCK)-8 assay

After treatments, cells were plated in 96-well plates at 5×10 ^3^ cells/well and cultured for 0, 12, 24, and 48 h. In each well, cells were incubated with 10 μL of CCK-8 reagent (Dojindo, Kumamoto, Japan) for 1 h according to the manufacturer’s protocol. Finally, the optical density was measured at 450 nm with a microplate reader (Bio-Rad, Hercules, USA).

### Flow cytometry of cell cycle

After treatments, cells were plated in 6-well plates at 2×10 ^5^ cells/well, centrifuged at 1000 *g* for 5 min, fixed with 700 μL pre-cooled absolute ethyl alcohol, incubated with 1 mg/mL of RNase A (100 μL) in the dark for 30 min and stained with 50 μg/mL of propidium iodide (PI; 400 μL) for 20 min. Finally, cells were analyzed by flow cytometry with a FACScan flow cytometer (Becton Dickinson, Franklin Lakes, USA) using Cell Quest software.

### Senescence β-galactosidase staining

After treatments, cells were washed with PBS for 3 times and stained with freshly prepared SA-β-Gal staining solution (Beyotime, Shanghai, China) according to the manufacturer’s protocol. The stained cells were examined under a microscope.

### Quantitative RT-PCR

Total RNA was extracted using Trizol reagent (Invitrogen) and the cDNA was synthesized by using a PrimeScript kit (TaKaRa, Dalian, China). RT-PCR was performed using SYBR green PCR master mix (Applied Biosystems, Foster City, USA) in an ABI 9700 real-time PCR system (Applied Biosystems). Fold-changes of mRNA expression were determined using the 2 ^−ΔΔCT^ method, and normalized to that of *GAPDH*. Primer sequences are listed in Table 1.

**Table:** **Table1** Sequence of primers used in this study

Gene	Primer sequence (5′→3′)
*USP1*	Forward Reverse	GATTATTTGCGGTTGTGATGC CATTCAATGGTTCTGGCTTACC
*USP4*	Forward Reverse	CTACTTTGGTTTGCCCAGAATG AGAGCCTCGCACAGGTCGG
*USP7*	Forward Reverse	ATGATGTTCAGGAGCTTTGTCG CCCCAGCGTCGTATTTATTGTC
*USP14*	Forward Reverse	CGAGAAAGGTGAACAAGGACAG TGCCGATGCAGATGAGGAG
*USP15*	Forward Reverse	GACGCTGCTCAAAACCTCG CTCCCATCTGGTATTTGTCCC
*USP20*	Forward Reverse	TGCGGAACGGAGTGAAGTAC AAGGAGACGTGGCTGTTGATC
*USP25*	Forward Reverse	ACACCCCACCAGAAACCG CCTATAATCCTGATGCCACTCC
*USP47*	Forward Reverse	TTTGCTACCTGAACAATCCCC GCTGCTGTCCCCATTATCTCC
*p53*	Forward Reverse	CCCCTCCTCAGCATCTTATCC ACAAACACGCACCTCAAAGC
*GAPDH*	Forward Reverse	AATCCCATCACCATCTTC AGGCTGTTGTCATACTTC

### Western blot analysis

Total protein was extracted using Radio Immunoprecipitation Assay (RIPA) lysis buffer (BYL40825; JRDUN Biotechnology, Shanghai, China). Proteins were separated by sodium dodecyl sulfate-polyacrylamide gel electrophoresis (SDS-PAGE) and transferred to polyvinylidene fluoride (PVDF) membrane (Millipore, Billerica, USA). The membrane was blocked with 5% skim milk and then incubated with primary antibodies against p53 (2524; Cell Signaling Technology, Danvers, USA), p21 (2947; Cell Signaling Technology), USP7 (ab4080; Abcam, Cambridge, USA,), or GAPDH (5174; Cell Signaling Technology), followed by incubation with the corresponding horse radish peroxidase (HRP)-conjugated secondary antibodies (A0208 and A0216; Beyotime). The content of protein was detected by using enhanced chemiluminescence reagents (Bio-Rad).

### Co-immunoprecipitation (Co-IP) and ubiquitination assay

Cell lysates were prepared using RIPA lysis buffer, incubated with anti-USP7 (ab264422; Abcam), anti-p53 (9282; Cell Signaling Technology,), or IgG antibody (Santa Cruz Biotech, Santa Cruz, USA), and then with Protein A/G PLUS-Agarose beads (Santa Cruz Biotech). The immunocomplexes were washed three times with lysis buffer, and then the ubiquitination level was detected by western blot analysis using anti-ubiquitin antibody (10201-2-AP; Proteintech, Rosemont, USA).

### Animal models

A total of 36 male SD rats (4–6 weeks, 180–200 g; Shanghai SLAC Laboratory Animal Co., Ltd., Shanghai, China)) were used in this study. They were randomized into three groups: in the control group, rats received saline; in the diabetic group, rats were intraperitoneally administrated with 60 mg/kg STZ citrate buffer to induce diabetes; and in diabetes+HBX 41108 group, rats received 60 mg/kg STZ citrate buffer and 100 mg/kg/day HBX 41108 (intraperitoneally injected once a day for 14 consecutive days). The diabetes model was established when the blood glucose of rats exceeded 16.7 mM after 48 h of STZ administration. The skin wounds (1.3 cm×1.3 cm) were made using a scissor on the midback of rats. Blood and wounds were detected on day 0, 7 and 14. All animal experiments were performed with the approval of the Institutional Animal Care and Use Committee of Qingpu Branch of Zhongshan Hospital.

### Hematoxylin and eosin (HE) staining

Wound tissues collected on day 0, 7 and 14 were fixed with 4% paraformaldehyde. HE staining was performed as previously described [19]. Digital images were captured at 200× magnification using an Olympus BX51 microscope equipped with an Olympus DP71 charge-coupled device camera (Olympus, Tokyo, Japan). Image analyses were performed by blinded operators.

### Statistical analysis

All statistical analyses were performed using GraphPad Prism 8.0.2. Experiments were conducted in triplicates and data were presented as the mean±standard deviation (SD). Group comparisons were performed by Student’s *t* test or one-way ANOVA followed by Tukey’s post-multiple test. *P*<0.05 was considered to be statistically significant.

## Results

### USP7 expression is increased in diabetic foot ulcer and in HUVECs after AGEs treatment

**Figure 1:**
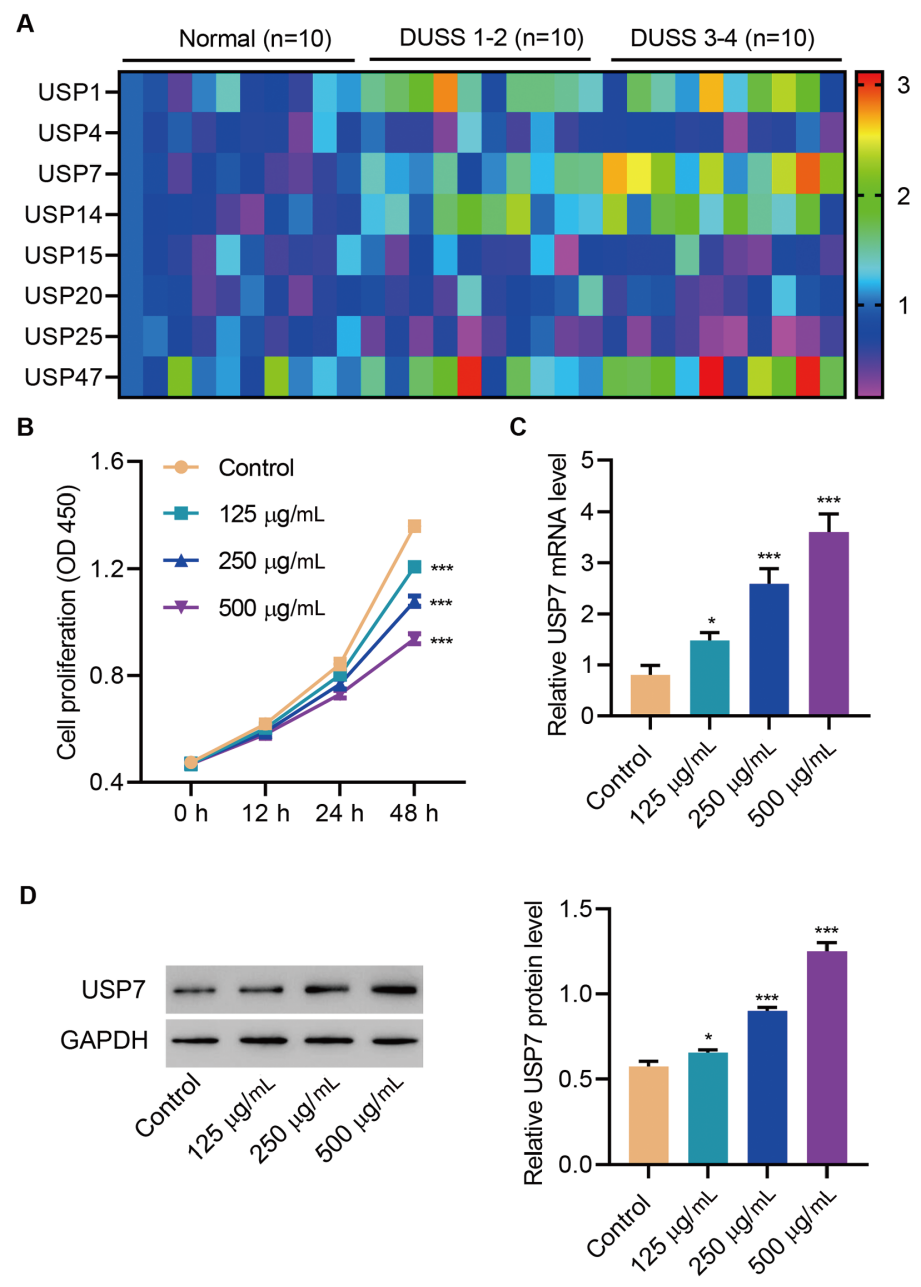
Figure1 **USP7 expression is increased in diabetic foot ulcer and HUVECs after AGEs treatment**(A) USP1, USP4, USP7, USP14, USP15, USP20, USP25 and USP47 mRNA expressions in normal skin tissues (n=10) and diabetic foot ulcers (n=20). (B–D) HUVECs were treated with AGEs. (B) Cell viability of HUVECs. The mRNA level (C) and protein level (D) of USP7 in HUVECs. *P<0.05, ***P<0.001 vs control.

To explore the role of USP family members in diabetic foot ulcer, we detected the mRNA expressions of several USP family members in diabetic foot ulcer with different DUSS and in normal skin tissues. USP7 expression was significantly elevated in diabetic foot ulcer tissues and further increased in lesions with high DUSS ( Figure 1A). AGEs were applied in HUVECs to mimic the situation of diabetes. CCK-8 assay revealed that AGEs significantly inhibited the proliferation of HUVECs in a dose-dependent manner ( Figure 1B). Meanwhile, the mRNA and protein levels of USP7 were significantly increased by AGEs ( Figure 1C,D). These results preliminarily demonstrated that USP7 might play a pathogenic role in diabetic foot ulcer.

### Inhibiting USP7 expression and its deubiquitinating activity reduces cell cycle arrest and cell senescence in HUVECs after AGEs treatment

**Figure 2:**
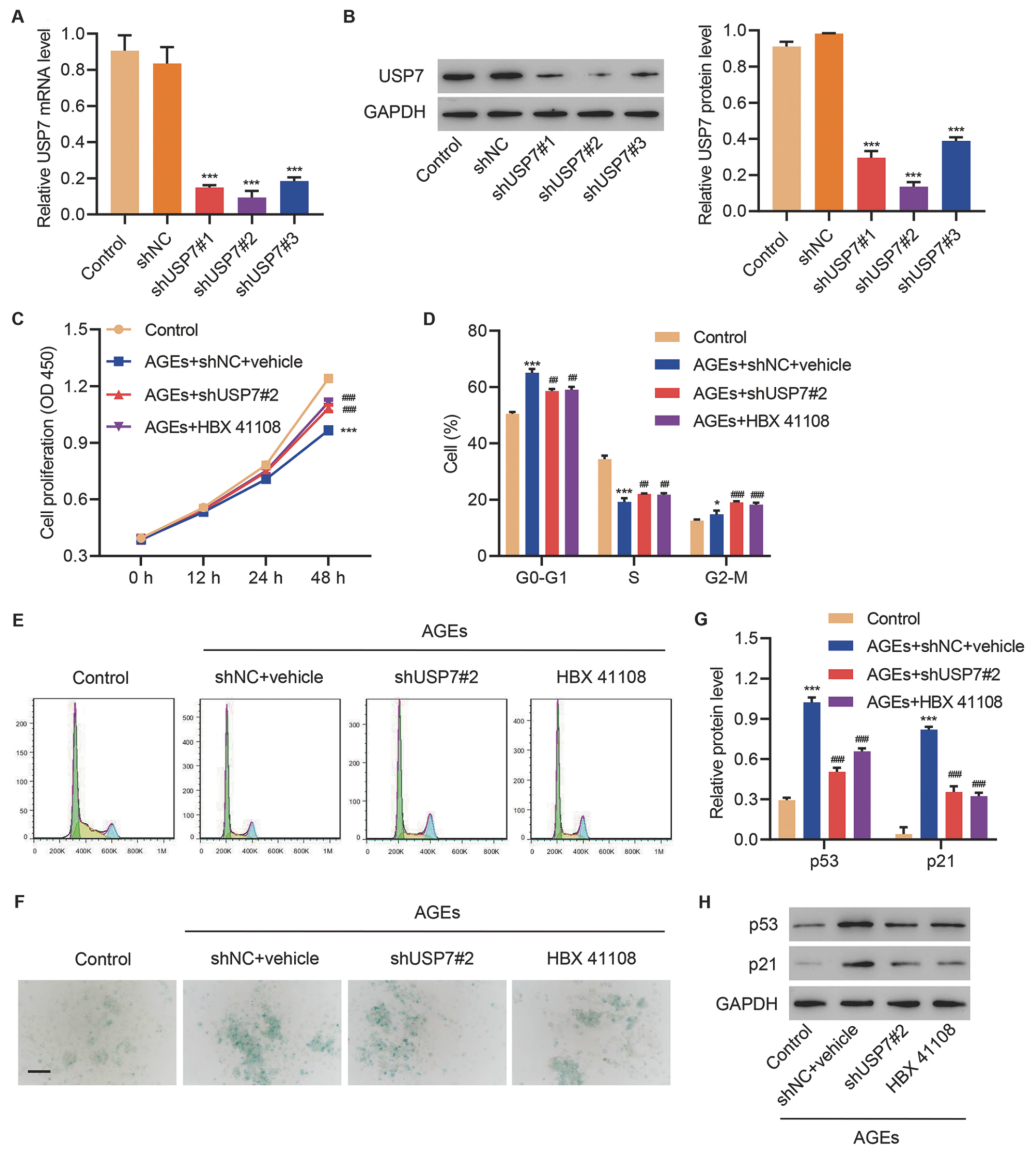
Figure2 I **nhibiting USP7 expression and its deubiquitinating activity reduces cell cycle arrest and cell senescence in HUVECs after AGEs treatment** (A,B) USP7 expression in HUVECs after the transduction of shUSP7 lentivirus. (C) Cell viability, (D,E) cell cycle, (F) cell senescence (scale bar=100 μm), and (G,H) expressions of p53 and p21 in HUVECs transduced with shUSP7 lentivirus or treated with USP7 inhibitor HBX 41108 (5 μM), following treatment with 250 μg/mL AGEs. *P<0.05, ***P<0.001 vs control. ##P<0.01, ###P<0.001 vs AGEs+shNC+vehicle.

To explore the biological function of USP7 in diabetic foot ulcer, we transduced corresponding shRNAs targeting USP7 in HUVECs treated with AGEs. The three shRNAs significantly inhibited the mRNA and protein levels of USP7 in HUVECs, and the shUSP7-2 exhibited the highest efficiency ( Figure 2A,B). The application of AGEs significantly reduced the proliferation of HUVECs, and the inhibition of USP7 by shUSP7 or HBX 41108 (a specific inhibitor of USP7 deubiquitinating activity) exerted opposite effects against AGEs ( Figure 2C). Moreover, the proportion of cells in the G0/G1 phase was significantly increased by AGEs, which was decreased by USP7 knockdown or HBX 41108, whereas that of cells in the S phase was significantly decreased by AGEs, which was increased by USP7 knockdown or HBX 41108 ( Figure 2D,E). In addition, AGEs markedly increased the level of cell senescence, whereas the inhibition of USP7 by shUSP7 or HBX 41108 could reverse this effect ( Figure 2F). p53 and p21 are markers of cell senescence. Western blot analysis revealed that the expressions of p53 and p21 were elevated by AGEs and decreased by shUSP7 and HBX 41108 ( Figure 2G,H). These results indicated that inhibition of USP7 expression and its deubiquitinating activity could reduce cell cycle arrest and cell senescence in HUVECs.

### USP7 interacts with p53 and mediates its deubiquitination

**Figure 3:**
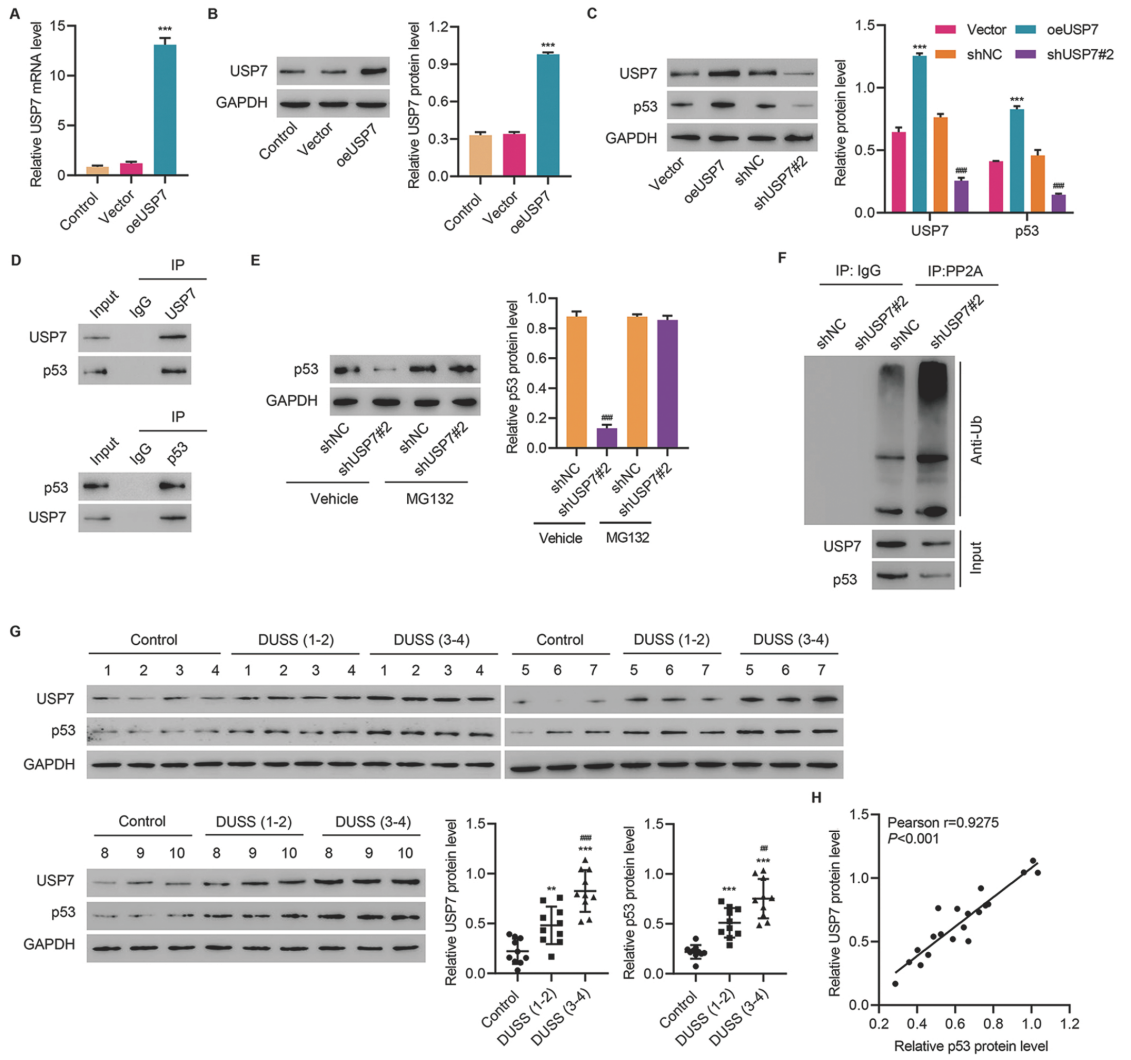
Figure3 **USP7 interacts with p53 and mediates its deubiquitination**(A–C) USP7 and p53 expressions in HUVECs transduced with oeUSP7 vector, blank vector, shUSP7, or shNC lentivirus vector. (D) Immunoprecipitation was performed using anti-USP7 or anti-p53 antibody. (E) p53 expression in HUVECs transduced with shUSP7 or shNC lentivirus and treated with 10 μM MG132 or vehicle. (F) The ubiquitination level of p53 in HUVECs transduced with shUSP7 lentivirus. (G) USP7 and p53 expressions in skin tissues of patients with diabetic foot ulcer (n=20) and normal skin tissues (n=10). (H) Pearson correlation plot of the expressions of p53 and USP7 in skin tissues (n=20). ***P<0.001 vs vector or control. ##P<0.01, ###P<0.001 vs shNC or DUSS.

USP7 has been demonstrated to mediate the deubiquitination of p53 in cancers [ 20, 21]. Therefore, we further explored the correlation between USP7 and p53 in HUVECs. Transduction of oeUSP7 lentivirus successfully increased the mRNA and protein levels of USP7 in HUVECs ( Figure 3A,B). Overexpression of USP7 markedly promoted the expressions of USP7 and p53, whereas knockdown of USP7 decreased the expressions of USP7 and p53 ( Figure 3C). Co-IP assay revealed that USP7 could interact with p53 ( Figure 3D). When the proteasome inhibitor, MG132, was applied in HUVECs, knockdown of USP7 failed to decrease the expression of p53, indicating the involvement of ubiquitination underlying the reduction of p53 ( Figure 3E). Moreover, Co-IP assay revealed that inhibition of USP7 could promote the ubiquitination level of p53 ( Figure 3F). Additionally, the expressions of USP7 and p53 were notably higher in diabetic foot ulcer skin specimens than those in the control group and further elevated in high-DUSS specimens ( Figure 3G). Pearson analysis revealed that the protein level of USP7 was positively associated with the protein level of p53 ( Figure 3H). These results indicated that USP7 could promote the expression of p53 by inducing its deubiquitination.

### P53 silencing ameliorates USP7 overexpression-mediated cell cycle arrest and cell senescence in HUVECs

**Figure 4:**
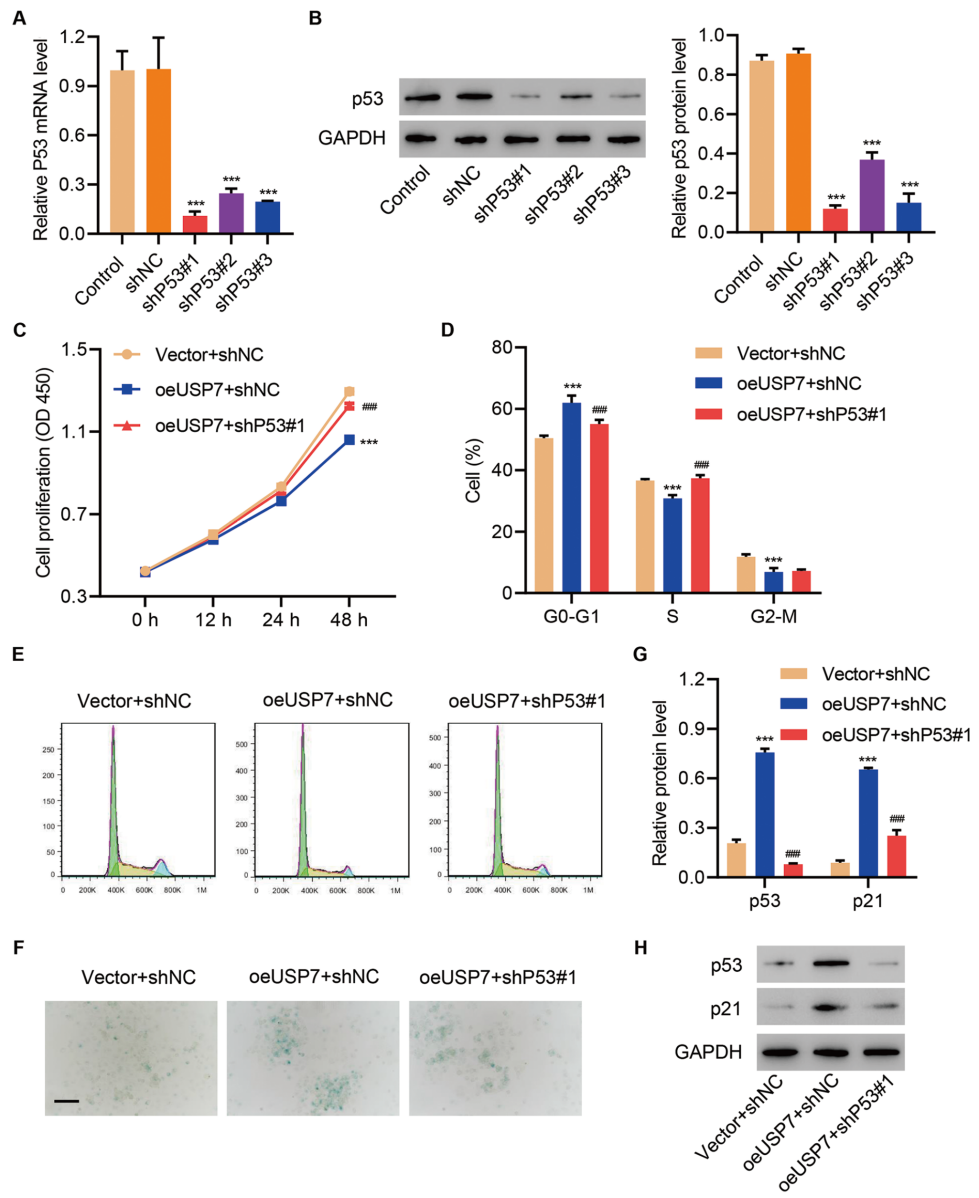
Figure4 **p53 silencing ameliorates USP7 overexpression-mediated cell cycle arrest and cell senescence in HUVECs**(A,B) p53 expression in HUVECs after the transduction with shP53 lentivirus. (C) Cell viability, (D,E) cell cycle, (F) cell senescence (scale bar=100 μm), and (G,H) the expressions of p53 and p21 in HUVECs transduced with oeUSP7 vector and shP53 lentivirus. ***P<0.001 vs shNC or vector+shNC. ###P<0.001 vs oeUSP7+shNC.

Then we verified the biological activities of USP7 and p53 in HUVECs. Three shRNAs targeting p53 were transfected into HUVECs, which significantly inhibited the mRNA and protein levels of p53 ( Figure 4A,B). Given the fact that shP53#1 exhibited the highest efficiency, we transduced the corresponding lentivirus into HUVECs. The proliferation rate of HUVECs was significantly suppressed by the overexpression of USP7 but rescued by the inhibition of p53 ( Figure 4C; *P*<0.05). Meanwhile, the proportion of cells in the G0/G1 phase was significantly promoted by oeUSP7 and decreased by shP53, whereas that of cells in the S phase was reduced by USP7 but increased by shP53 ( Figure 4D,E). Moreover, the inhibition of p53 could reverse the elevated level of cell senescence mediated by USP7 ( Figure 4F). Additionally, the expressions of p53 and p21 could be promoted by the overexpression of USP7, which was reversed by the downregulation of p53 ( Figure 4G,H). These results suggested that p53 silencing could reverse USP7-mediated cell cycle arrest and cell senescence in HUVECs.

### HBX 41108 facilitates wound healing in diabetic rats

**Figure 5:**
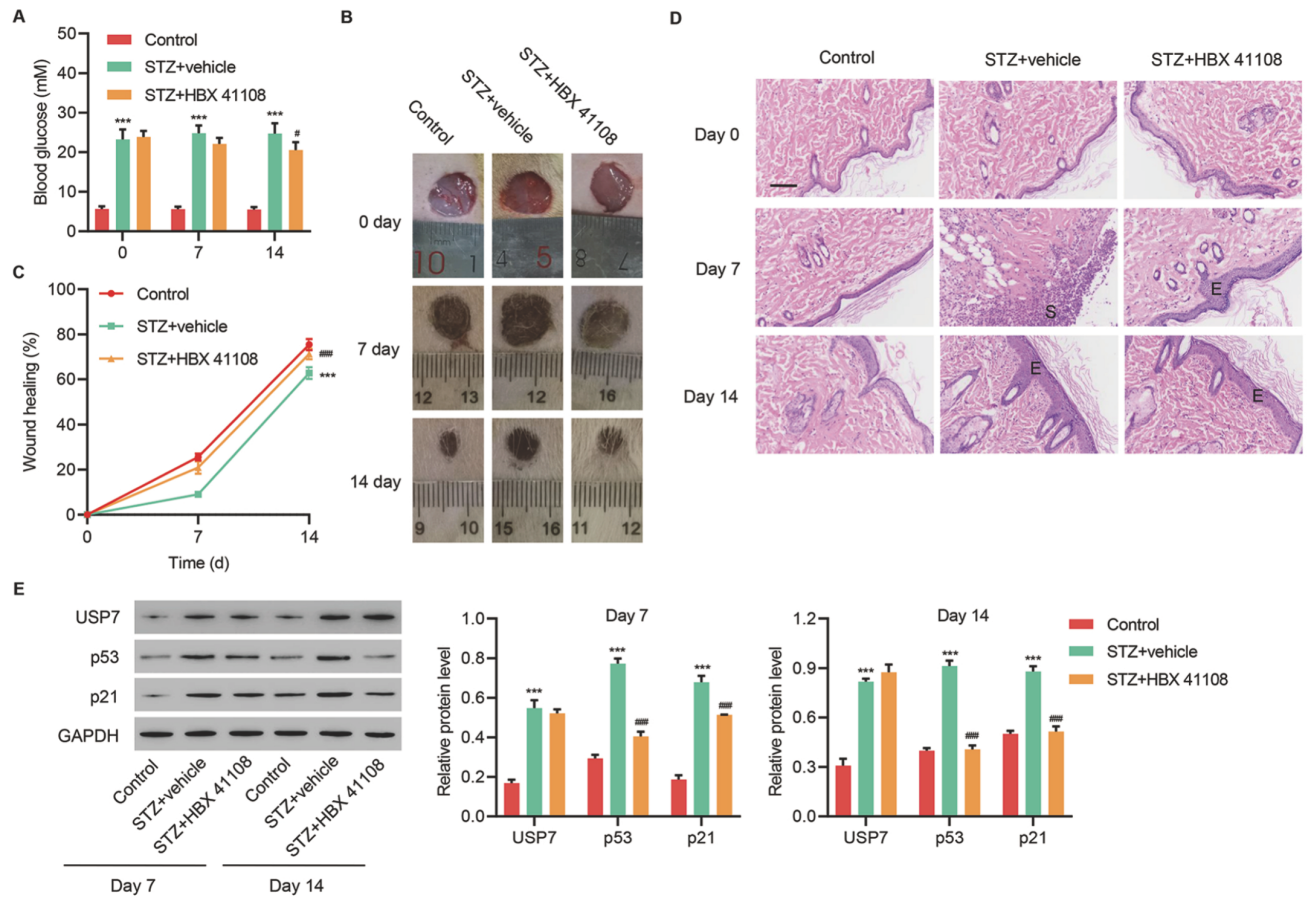
Figure5 **HBX 41108 facilitates wound healing in diabetic rats Rats were divided into control group, the STZ group, and HBX 41108 group**(A) Blood glucose level (mM) changes in different groups of rats. (B) The images of skin wounds at day 0, 7, and 14 post-injury. (C) The wound healing rates at day 0, 7, and 14. (D) HE staining of wound tissues (scale bar=100 μm). (E) Expressions of USP7, p53 and p21 in tissues at day 7 and 14 post-injury in each group. ***P<0.001 vs control. #P<0.05, ###P<0.001 vs STZ+vehicle. S, scab; E, epidermis.

We further validated the therapeutic effects of HBX 41108 *in vivo*. Rats were intraperitoneally administrated with 60 mg/kg STZ citrate buffer to establish a diabetic rat model. Then, HBX 41108 (100 mg/kg/day) was intraperitoneally administrated once a day for 14 days. Results showed that untreated diabetic rats (STZ) had higher blood glucose levels than nondiabetic rats (control) on day 0, 7 and 14 post-injury. Treatment of diabetic rats with HBX 41108 decreased blood glucose levels on 14 post-injury ( Figure 5A). STZ significantly reduced the wound healing rate, whereas the application of HBX 41108 could rescue the STZ-mediated effects ( Figure 5B,C). HE staining of tissue sections showed that epithelial tissue was newly formed in wounds treated with HBX 41108 on day 7 post-injury ( Figure 5D). Meanwhile, on day 7 and day 14, the expressions of USP7, p53, and p21 were found to be increased by STZ and decreased by the application of HBX 41108 ( Figure 5E). These results showed that HBX 41108 could facilitate wound healing in diabetic rats through inhibiting USP7 deubiquitinating activity.

## Discussion

Diabetic foot is a long-term complication of diabetes that intensively impairs the quality of patients’ lives. Current management of diabetic foot remains a challenge and requires long-term management. Herein, we found that USP7 is highly expressed in patients with diabetic foot ulcer and in HUVECs after AGEs treatment. Inhibition of USP7 could reduce cell cycle arrest and cell senescence in HUVECs. Moreover, USP7 could increase the expression of p53 through promoting its deubiquitination. As a specific inhibitor of USP7 deubiquitinating activity, HBX 41108 could significantly accelerate wound healing in diabetic rats. Our study revealed a novel signaling pathway underlying the pathogenesis of diabetic foot and provided a potential strategy to treat diabetic foot. USP7 was found to be highly expressed in diabetic foot ulcers with high DUSS. Meanwhile, a previous study indicated that AGEs could promote cell senescence and induce oxidative stress in human trabecular meshwork cells [22]. Furthermore, the receptor of AGEs was shown to be one of the targets of metformin in the treatment of diabetes [23]. Therefore, AGEs were applied to establish diabetic cell model and the expression of USP7 was promoted by AGEs. USP7 is a member of USP family that contains a multitude of deubiquitinases. Accumulating studies suggested that USP7 is closely associated with the pathogenesis of various diseases, such as neurodegenerative diseases [24], myocardial ischemia [25] and cancer [26].

Although the tumor suppressing activity of p53 has been well characterized, p53 was also shown to be involved in other diseases such as diabetes [27]. Minamino et al. [28] revealed that p53 could lead to insulin resistance and increase the risk of diabetes in mice. Knockout of p53 could ameliorate cell senescence and reduce inflammation in adipose tissues of mice, which avoided the development of insulin resistance [28]. Moreover, p53-mediated senescence would diminish self-replication of pancreatic beta cell and severe diabetes [29]. In addition, as the downstream of p53 and a regulator of cell cycle, p21 was reported to regulate the regeneration capacity of pancreatic beta cells [30]. Therefore, p53 and p21 are promising targets for the treatment of diabetes. In this study, we showed that inhibition of p53 expression significantly promoted the proliferation of HUVECs and reduced cell senescence. Meanwhile, inhibition of p53 expression significantly reduced cell cycle arrest. Therefore, our study indicated that p53 is a potential target for the treatment of diabetic foot ulcers.

It has previously been reported that USP7 is a gene required to maintain a healthy lifespan, and lifespan is shortened when the USP7 is knockdown in *Drosophila* and *Caenorhabditis elegans* [ 31, 32]. In this study, HBX 41108, a cyanoindenopyrazine-derived compound that acts as a potent, reversible, and substrate competitive USP7 inhibitor [33], was used to investigate the influence of USP7 deubiquitinase activity on AGEs-induced cell cycle arrest and senescence of HUVECs. Inhibition of USP7 deubiquitinating activity using HBX 41108 could activate and stabilize p53 to inhibit the proliferation of cancer cells [34]. Knockdown of USP7 can also activate p53 signaling and lead to cell cycle arrest [20], which makes USP7 as a therapeutic target due to its activation of p53 [26]. It has been reported that inhibition of USP7 can eliminate senescent cells [35], which is in line with our finding that there is a clear decrease in p53 expression after HBX 41108 treatment or USP7 silencing, suggesting that USP7 may directly or indirectly regulate p53 expression and/or stability under different pathological conditions. Since p53 is highly associated with diabetic foot ulcers, USP7 may be a potential target for the treatment of diabetic foot ulcers. In addition to p53, p21 has also been reported to be involved in the decreased cell growth of HUVECs induced by AGEs via modulation of MEG3/miR-93 [36]. Thus, AGE-induced p53 and/or p21 might be a novel regulatory network in diabetic vascular cells, and possess potential therapeutic value for diabetes mellitus.In summary, our study revealed that inhibition of USP7 could suppress AGEs-induced cell cycle arrest and cell senescence of HUVECs through promoting p53 ubiquitination. USP7 may be a promising target for the treatment of diabetic foot ulcers.
